# Self-radiolysis of tritiated water. 4. The scavenging effect of azide ions (N_3_^−^) on the molecular hydrogen yield in the radiolysis of water by ^60^Co γ-rays and tritium β-particles at room temperature

**DOI:** 10.1039/c7ra12397c

**Published:** 2018-01-12

**Authors:** Sunuchakan Sanguanmith, Jintana Meesungnoen, Craig R. Stuart, Patrick Causey, Jean-Paul Jay-Gerin

**Affiliations:** Département de médecine nucléaire et de radiobiologie, Faculté de médecine et des sciences de la santé, Université de Sherbrooke 3001, 12^e^ Avenue Nord Sherbrooke Québec J1H 5N4 Canada jean-paul.jay-gerin@USherbrooke.ca; Reactor Chemistry and Corrosion Branch, Canadian Nuclear Laboratories Chalk River Ontario K0J 1J0 Canada; Radiological Protection Research and Instrumentation Branch, Canadian Nuclear Laboratories Chalk River Ontario K0J 1J0 Canada

## Abstract

The effect of the azide ion N_3_^−^ on the yield of molecular hydrogen in water irradiated with ^60^Co γ-rays (∼1 MeV Compton electrons) and tritium β-electrons (mean electron energy of ∼7.8 keV) at 25 °C is investigated using Monte Carlo track chemistry simulations in conjunction with available experimental data. N_3_^−^ is shown to interfere with the formation of H_2_ through its high reactivity towards hydrogen atoms and, but to a lesser extent, hydrated electrons, the two major radiolytic precursors of the H_2_ yield in the diffusing radiation tracks. Chemical changes are observed in the H_2_ scavengeability depending on the particular type of radiation considered. These changes can readily be explained on the basis of differences in the initial spatial distribution of primary radiolytic species (*i.e.*, the structure of the electron tracks). In the “short-track” geometry of the higher “linear energy transfer” (LET) tritium β-electrons (mean LET ∼5.9 eV nm^−1^), radicals are formed locally in much higher initial concentration than in the isolated “spurs” of the energetic Compton electrons (LET ∼0.3 eV nm^−1^) generated by the cobalt-60 γ-rays. As a result, the short-track geometry favors radical–radical reactions involving hydrated electrons and hydrogen atoms, leading to a clear increase in the yield of H_2_ for tritium β-electrons compared to ^60^Co γ-rays. These changes in the scavengeability of H_2_ in passing from tritium β-radiolysis to γ-radiolysis are in good agreement with experimental data, lending strong support to the picture of tritium β-radiolysis mainly driven by the chemical action of short tracks of high local LET. At high N_3_^−^ concentrations (>1 M), our H_2_ yield results for ^60^Co γ-radiolysis are also consistent with previous Monte Carlo simulations that suggested the necessity of including the capture of the precursors to the hydrated electrons (*i.e.*, the short-lived “dry” electrons prior to hydration) by N_3_^−^. These processes tend to reduce significantly the yields of H_2_, as is observed experimentally. However, this dry electron scavenging at high azide concentrations is not seen in the higher-LET ^3^H β-radiolysis, leading us to conclude that the increased amount of intra-track chemistry intervening at early time under these conditions favors the recombination of these electrons with their parent water cations at the expense of their scavenging by N_3_^−^.

## Abstract 2

The effect of the azide ion on the yield of molecular hydrogen in water irradiated with ^60^Co γ-rays and tritium β-electrons at 25 °C is investigated using Monte Carlo track chemistry simulations.

## Graphical abstract

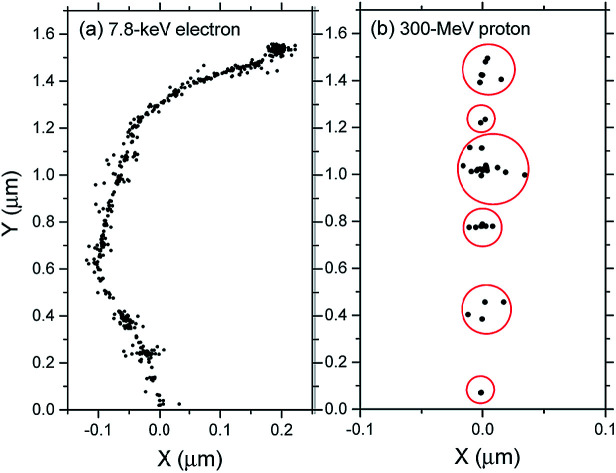

## 1. Introduction

**Fig. 1:**
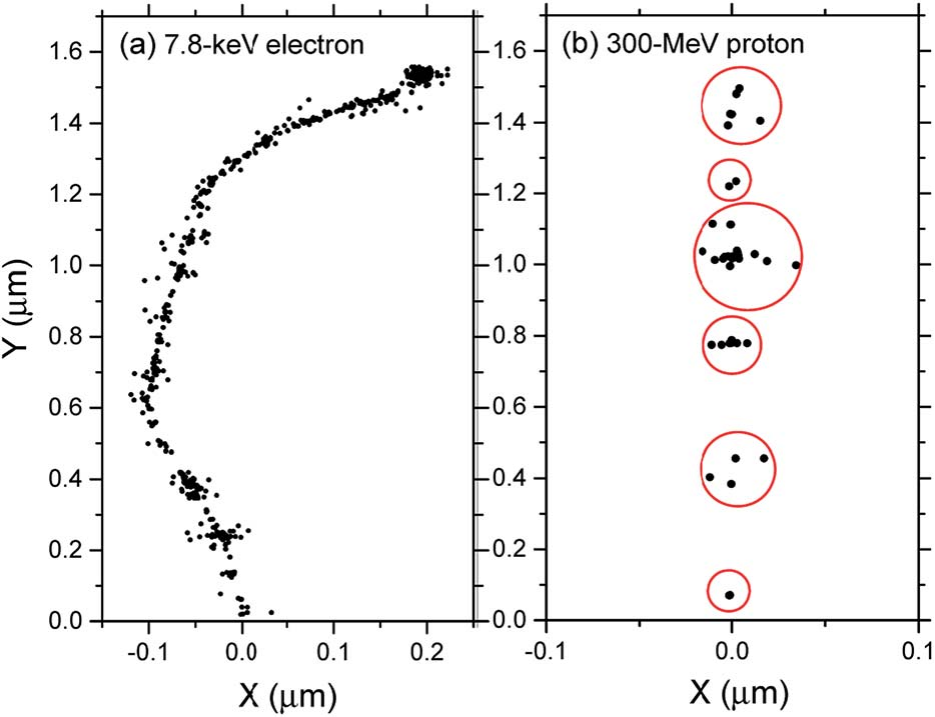
Simulated track histories (projected into the *XY* plane of figure) of a 7.8 keV tritium β-electron (complete track; mean LET ∼ 5.9 eV nm^−1^) (panel a) and a 300 MeV proton (track segment; LET ∼ 0.3 eV nm^−1^) (panel b) incident in liquid water at 25 °C. The two irradiating particles are generated at the origin and start traveling along the *Y* axis. Dots represent the energy deposited at points where an interaction occurred.

A detailed understanding of the radiolysis of water and aqueous solutions is important both from a fundamental science point of view and for a variety of practical applications,^1–4^ in particular, in the nuclear power industry and in radiation biology where living cells and tissues consist mainly of water (∼70–85% by weight). Exposed to ionizing radiation, water is the site of ionizations and excitations:
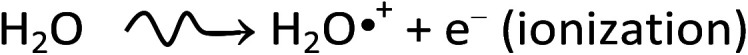

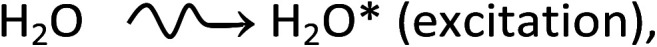
which result, within a few picoseconds, in a cascade of events leading to the formation of free radicals and molecular products along the track of the incident radiation. Ejected secondary electrons (also called “dry” electrons) have generally sufficient kinetic energy to cause further ionizations and excitations in close proximity to the original water positive ion. After slowing down to sub-excitation energies and thermalization, these electrons become trapped and hydrated. Under ordinary irradiation conditions (*i.e.*, at modest dose rates so that no track overlap occurs), the initial products of radiolysis are generated in a highly nonhomogeneous “track structure” geometry.^5–11^ They include^12,13^ the hydrated electron (e_aq_^−^), H_3_O^+^, OH^−^, H˙, H_2_, ˙OH, H_2_O_2_, O_2_˙^−^ (or its protonated form HO_2_˙; p*K*_a_ = 4.8 at 25 °C), O(^1^D), ˙O˙(^3^P), O˙^−^, *etc.* This early nonhomogeneous spatial distribution of radiolytic species is strongly dependent on the radiation quality, a measure of which is given by the “linear energy transfer” (LET) (also called “stopping power” by physicists and denoted by −d*E*/d*x*). For low-LET, sparsely ionizing radiation (such as γ-rays from ^60^Co or fast electrons; LET ∼0.3 eV nm^−1^), tracks are formed initially by widely spaced clusters of reactive species, commonly known as “spurs” (spherical in shape).^14,15^ In this case, the predominant effect of radiolysis is radical production. In fact, when diffusion has brought about homogeneity in the system (*i.e.*, within a few microseconds after the initial energy deposition), relatively few radicals have combined in the spurs, resulting in an excess of radicals over molecular products. However, with increasing LET, the isolated spur structure changes to a situation in which the spurs eventually overlap and form (initially) a dense continuous column of species. This is actually the case for the low-energy β-electrons of tritium, which are involved in the “self-radiolysis” of tritiated water (^3^HOH),^16–18^ the subject matter of the present study. In the terminology of the Mozumder–Magee model of energy deposition,^6,19^ while the Compton electrons (∼1 MeV) produced by ^60^Co γ-radiolysis predominantly form spurs, these soft, higher-LET tritium β-electrons predominantly deposit their energy as “short tracks”. This leads to an increased local concentration of reactants and therefore an increased amount of intra-track chemistry that favors radical–radical reactions. Under these conditions, the radiation chemical yields (or *G*-values)^20^ of the molecular products increase at the expense of the individual radicals. For the sake of illustration, Fig. 1 shows typical 2-D representations of the complete track of a 7.8 keV ^3^H β-electron and the track segment of a 300 MeV proton (which mimics irradiation with ^60^Co γ-rays), calculated with our IONLYS Monte Carlo track structure simulation code (see below).

In close connection with the LET and the relationship between track structure and chemistry, one critical area of research focuses on elucidating the basic radiation chemical mechanisms that operate in the “self-radiolysis” of tritiated water as compared with ^60^Co γ-radiolysis.^21–26^ The present paper is the fourth in a series^18,27,28^ dedicated to this subject. The Monte Carlo track-chemistry simulation work we reported previously revealed significant differences between the chemical properties of short tracks and spurs using either γ-rays/fast electrons or tritium β-particles. Overall, the results of our simulations provided strong support for the picture of tritium β-radiolysis mainly driven by the chemical action of short tracks of high local LET. In the present study, we now attempt to distinguish further the chemical properties of spur and short track geometries by examining the differences in the scavengeability of molecular H_2_ – whose yields are relatively well-documented experimentally^21,22,25,26,29–32^ – when passing from γ- to tritium β-electron radiolysis.

Molecular hydrogen is one of the most interesting radiolytic species, in part because of the questions it raises about the source of its formation. At very short times (<50–300 fs) after the passage of the ionizing radiation,^33^ H_2_ can be formed by the following reactions:^34,35^

(1) geminate recombination of the sub-excitation electron (e_sub_^−^)^36^ with its parent cation H_2_O˙^+^H_2_O˙^+^ + e_sub_^−^ → H_2_O*

(2) “dissociative electron attachment” (or DEA) involving the resonant capture of e_sub_^−^ by a water moleculeH_2_O + e_sub_^−^ → (H_2_O˙^−^*) → H^−^ + ˙OH,

followed byH^−^ + H_2_O → H_2_ + OH^−^

(3) dissociation of excited water molecules (formed either by direct excitation or by geminate electron-hole recombination)H_2_O* → H_2_ + O(^1^D),where O(^1^D) is the oxygen atom in its singlet ^1^D first excited state.

In the low-LET γ-irradiation case, this “initial” – then described as “unscavengeable” (*i.e.*, not removable by scavenger experiments) – H_2_ yield was first estimated by Schwarz^37^ in 1969 to be ∼30% of the total “escape” yield^20^ for molecular hydrogen [*g*(H_2_) = 0.45 molecule/100 eV].^5,7,11,12^ Recent scavenger studies^38^ have shown, however, that Schwarz's initial estimate was undervalued. In fact, it was found that a major fraction (∼75%, *i.e.*, ∼0.34 molecule/100 eV) of the total H_2_ produced was due to reactions of the dry/subexcitation electrons in the subpicosecond physicochemical stage of the radiolysis. In other words, these results suggest that only ∼0.11 molecule of H_2_ per 100 eV remains to be formed during the subsequent nonhomogeneous chemical stage (*i.e.*, in the radiation tracks as they expand by diffusion) on the picosecond–microsecond time scale. At this stage, three radical–radical combination reactions of the hydrated electron and H˙ atom intervene in hydrogen formation. They are^18,34,39,40^

1 e_aq_^−^ + H˙ (+H_2_O) → H_2_ + OH^−^, *k*_1_ = 2.5 × 10^10^ M^−1^ s^−1^

2 e_aq_^−^ + e_aq_^−^ (+2H_2_O) → H_2_ + 2OH^−^, *k*_2_ = 6.2 × 10^9^ M^−1^ s^−1^

and, but to a much lesser extent,

3 H˙ + H˙ → H_2_, *k*_3_ = 4.6 × 10^9^ M^−1^ s^−1^

with the corresponding rate constants (*k*) taken from the compilation of Elliot and Bartels.^12^

Besides the mechanism of its formation, a better knowledge of the radiolytic production of molecular hydrogen is crucial in the “water chemistry” management of current water-cooled nuclear reactors to optimize plant performance and lifetime. As we know,^2^ H_2_ is currently added to the primary coolant water to suppress the formation of stable oxidizing products (H_2_O_2_ and eventually its decomposition product O_2_) from water radiolysis by a short chain reaction, thereby preventing corrosion and activity transport. The *in situ* radiolytic formation of H_2_ in these reactors could, therefore, affect the minimum concentration of excess H_2_, referred to as the “critical hydrogen concentration”,^41,42^ required to suppress net radiolysis (no stable products formed) in the cores. Knowledge of this optimum H_2_ level, which would minimize the damaging consequences of corrosion, is still a subject of debate in the chemical literature.

The anomalous increase in the escape yield of H_2_ at high temperature is another key motivation for this study. In fact, although H_2_ is a molecular product, *g*(H_2_) increases with temperature under γ/fast electron irradiation,^12,34,35^ from ∼0.45 molecule/100 eV at 25 °C to ∼0.76 molecule/100 eV at 350 °C. This behavior is an exception to the generally accepted diffusion-kinetic model,^6,43^ which predicts that, when the temperature increases, diffusion of free radicals out of spurs or tracks becomes more important than recombination, resulting in less molecular recombination products. At present, no definitive mechanism has yet been established to account for this anomalous radiolytic production of H_2_ at high temperature.^34^

For these different reasons, the escape yield of H_2_ has attracted much attention from experimentalists and modelers in order to explore in more detail its formation under various irradiation conditions. In this work, we use Monte Carlo track chemistry simulations to examine further the chemical differences underlying the production of molecular hydrogen in tritium β-radiolysis as compared with cobalt γ-radiolysis. No real-time studies on H_2_ formation have been performed; its temporal dependence is usually probed by varying the concentration of appropriate scavengers for the hydrated electron and the hydrogen atom, which are the dominant free radical precursors of H_2_ within the diffusing spurs or tracks. We here report data on the scavengeability by azide ions (N_3_^−^) of the molecular H_2_ yield produced by γ- and tritium β-radiolysis. This particular scavenger was chosen as it presents very different reactivities towards e_aq_^−^ and H˙ atoms, being highly unreactive towards the former but reacting very rapidly with the latter. Our aim is to study the different H_2_ scavengeabilities found for the two types of irradiation considered and to examine how these differences reflect the structure of the radiation track (*i.e.*, spurs *vs.* short tracks) in both cases.

## 2. Monte-Carlo track chemistry simulations

Monte Carlo simulation methods are well suited to take into account the stochastic nature of the complex sequence of events that are generated in irradiated aqueous solutions containing reactive scavengers. In the case of interest here, the experimentally observed yield value for molecular hydrogen is a composite one to which each of the processes producing H_2_ contributes. The addition of a scavenger that competes with these processes to different extents will change the relative amount that each process contributes to the total yield. The simulation allows the reconstruction of the intricate action of the radiation, thus providing a powerful tool for studying the relationship between the initial radiation track structure, the ensuing chemical processes, and the stable final products formed. In this work, a full Monte Carlo track-chemistry computer code, called IONLYS-IRT,^11^ has been used to simulate the radiolysis of water and aqueous solutions containing various concentrations of scavengers. This code first models, in a 3D geometrical environment, the initial, highly nonhomogeneous radiation track structure (“IONLYS” program), and then the diffusion and chemical reactions of the various radical and molecular products formed by radiolysis with themselves or with solutes if present (“IRT” program). A detailed description of this code has been given previously.^11,18,28,34,44–46^ Only a brief overview of its most essential features is given below.

The IONLYS code is a step-by-step simulation program that covers the early physical and physicochemical stages^47^ of radiation action up to ∼1 ps in the track development. It is composed of two modules. One is for transporting the investigated incident charged particle (called either TRACEPR for an impacting primary electron or TRACPRO/TRACION for an incident proton/ion). The other (called TRACELE) is for transporting all of the energetic (or dry) electrons (collectively named “secondary electrons”) resulting from the ionization of the water molecules until they become hydrated. In this study, we used the TRACEPR module of IONLYS to simulate the track structures of low-energy (∼7.8 keV) tritium β-electrons. As for the TRACPRO module, it was used here to simulate track segments of 300 MeV incident protons (which, as mentioned before, mimic ^60^Co γ/fast electron irradiation) (see Fig. 1).

The complex, highly nonhomogeneous spatial distribution of reactants at the end of the physicochemical stage is provided as an output of the IONLYS (TRACELE) program. It is then used directly as the starting point for the subsequent nonhomogeneous chemical stage^47^ (from ∼1 ps to ∼0.1–1 μs at 25 °C,^48^*i.e.*, until all tracks/spurs have dissipated). This stage, during which all different species diffuse (we assume ∼1 ps also marks the beginning of diffusion) randomly at rates determined by their diffusion coefficients and react with one another or with any added solutes present at the time of irradiation, is covered by our “independent reaction times” (IRT) program. This program employs the IRT method,^49,50^ a computer-efficient stochastic simulation technique used to simulate reaction times without having to follow the trajectories of the diffusing species. Its implementation has been previously described in detail^45^ and its ability to give accurate, time-dependent chemical yields has been well validated^51,52^ by comparison with full random flight (or step-by-step) Monte Carlo simulations, which do follow the reactant trajectories in detail. Finally, this IRT program has also been used successfully to describe the evolution of radiation-induced yields in the homogeneous chemical stage^47^ after spur/track expansion is complete (*i.e.*, when the radiolytic products become homogeneously distributed in the bulk solution), in the time domain typically beyond a few microseconds.

The reaction scheme and rate constants for the radiolysis of pure liquid water at 25 °C employed in the current version of IONLYS-IRT are the same as used previously (see Table 1 of ref. 18). The values of the diffusion coefficients of the various intervening track species are listed in Table 6 of ref. 53. In order to simulate the radiolysis of the N_3_^−^ solutions, we have supplemented the pure-water reaction scheme to include the primary e_aq_^−^ and H˙ atom scavenging reactions that occur in the system (*vide infra*). Under normal irradiation conditions, the concentrations of radiolytic products are low compared with the background concentrations of N_3_^−^ ions considered, and their reactions could be modeled in the IRT program as pseudo first-order reactions. In the computer simulations reported here, the diffusion coefficient used for N_3_^−^ in liquid water at 25 °C was 1.84 × 10^−5^ cm^2^ s^−1^.^54^ This same value was also used for the diffusion coefficient of the azide radical N_3_˙.

In addition, we have introduced in the IRT program the effect of the ionic strength of the solutions on all reactions between ions.^55^ The correction to the reaction rate constants was made as described in ref. 56 and 57. Finally, for highly concentrated N_3_^−^ solutions (some experimental data are available up to 5 M), we neglected complications due the “direct” action of ionizing radiation on the solute (which our Monte Carlo code does not take into account). This is certainly a very good approximation for ∼1–2 M N_3_^−^ concentrations (in that case, ∼2–4% of the total energy is absorbed directly by the azide anions). For 5 M N_3_^−^ solutions, the proportion of direct effects increases to about 11%, which remains relatively low and may reasonably still be ignored at least as a first approximation.

To mimic the effects of ^60^Co γ/fast electron-radiolysis, we used short segments (typically, ∼150 μm) of ∼300 MeV irradiating proton tracks, over which the average LET of the proton remains nearly constant and equal to ∼0.3 eV nm^−1^ at 25 °C.^2,45^ Such model calculations thus gave “track segment” yields^8,45^ at a well-defined LET. Briefly, the simulations, performed with the TRACPRO module of IONLYS, consisted of following the transport and energy loss of an incident proton until it penetrated the chosen length of the track segment into the solution. As shown in Fig. 1, due to its large mass, the impacting proton is almost not deflected by collisions with the target electrons. The number of individual proton “histories” (usually ∼150) was chosen to ensure only small statistical fluctuations in the computed averages of chemical yields, while keeping acceptable computer time limits.

As indicated above, tritium-β primary electron track structures were simulated using the TRACEPR module of IONLYS. Each simulation typically involved 6000 different whole track histories. This number was chosen to permit averaging of results with acceptable statistical confidence. In all the simulations, a single “effective” initial electron energy of ∼7.8 keV (mean LET in water: ∼5.9 eV nm^−1^)^16^ was used to mimic the radiation chemical action of the tritium β-particles at 25 °C (Fig. 1). This energy was found previously to be better suited to produce representative *G*-values when using tritium β-rays than the commonly used mean kinetic energy of ∼5.7 keV released by tritium decay.^18,58^

Throughout this study, we assumed that tritiated water could simply be described as a “dilute” solution of ^3^HOH in light water, with concentrations of low volumic activity (typically, less than ∼1 Ci per mL) so that dose-rate effects could be ignored.^25,59^

## 3. Results and discussion

The azide ion N_3_^−^ reacts very fast with H˙ atoms and very slowly with the hydrated electron, according to^22,25,60–63^

4 N_3_^−^ + H˙ → HN_3_˙^−^, *k*_4_ = 3.15 × 10^9^ M^−1^ s^−1^

5 N_3_^−^ + e_aq_^−^ → products, *k*_5_ ≤ 1.5 × 10^6^ M^−1^ s^−1^,

where the decay of HN_3_˙^−^ by proton addition has been shown not to involve H_2_ as a final product^63^ and where it is assumed here that the products of reaction (5) do not influence the H_2_ chemistry. In contrast, its protonated form, hydrazoic acid (or hydrogen azide) HN_3_,^22,54,63^

6 N_3_^−^ + H^+^ ↔ HN_3_, p*K*_a_(HN_3_/N_3_^−^) = 4.7 in water at 25 °C, *k*_6_(forward) ≈ 10^9^–10^10^ M^−1^ s^−1^,

is highly reactive towards e_aq_^−^ but it reacts slower with H˙ atoms:^60,62–64^

7 HN_3_ + e_aq_^−^ → HN_3_˙^−^, *k*_7_ = 1.2 × 10^10^ M^−1^ s^−1^

8 HN_3_ + H˙ → products (≠H_2_), *k*_8_ = 6.3 × 10^7^ M^−1^ s^−1^

However, even if a fraction of the azide ions may react with H^+^ ions in the spurs/tracks^13,22^ to yield HN_3_, especially at high N_3_^−^ concentration (which is equivalent to short times), the Henderson–Hasselbalch equation indicates that, under the neutral pH conditions of this work, this compound will exist almost entirely in anion form. Hence, HN_3_ should not significantly affect the radiolytic H_2_ yield.

The azide ion can also react with the ˙OH radical to produce the one-electron oxidant azide radical, N_3_˙:^64,65^

9 N_3_^−^ + ˙OH → N_3_˙ + OH^−^, *k*_9_ = 1.2 × 10^10^ M^−1^ s^−1^,

or, for its protonated form,^64^

10 HN_3_ + ˙OH → N_3_˙ + H_2_O, *k*_10_ < 10^7^ M^−1^ s^−1^.

In this case, the ˙OH radicals (or at least part of them) are replaced by the N_3_˙ radicals and we need to consider the following reactions:^60,62,64,66–71^

11 N_3_˙ + e_aq_^−^ → N_3_^−^, *k*_11_ = 2.4 × 10^10^ M^−1^ s^−1^

12 N_3_˙ + H˙ → HN_3_, *k*_12_ ≈ 10^10^ M^−1^ s^−1^

13 N_3_˙ + H_2_O_2_ → products, *k*_13_ < 5 × 10^6^ M^−1^ s^−1^

14 N_3_˙ + N_3_˙ → 3N_2_, *k*_14_ = 3.9 × 10^9^ M^−1^ s^−1^

15 N_3_˙ + N_3_^−^ → (N_3_)_2_˙^−^, *k*_15_ = 2.4 × 10^5^ M^−1^ s^−1^

The rather slow reaction of azide with e_aq_^−^ virtually excludes any effect of N_3_^−^ on reactions involving e_aq_^−^ in the spurs/tracks,^60^ particularly in solutions with low N_3_^−^ concentration. Indeed, even in a 5 M N_3_^−^ solution, the scavenging time^72^ of e_aq_^−^ by N_3_^−^ is about the same order of magnitude as the lifetime of a spur (∼0.2 μs)^48^ in the ^60^Co γ-radiolysis of water at 25 °C. Under these conditions, the molecular hydrogen yield was measured in irradiated *aerated* azide solutions.^22,25,60^ Oxygen between ∼2.5 × 10^−4^ M (air-saturated conditions) and ∼3–5 × 10^−5^ M was used as e_aq_^−^ scavenger on the ∼0.1–1 μs time scale. Noteworthy, the azide radical is inert towards molecular oxygen,^66^ but may react with the superoxide anion radical^62,66^

16 N_3_˙ + O_2_˙^−^ → O_2_ + N_3_^−^, *k*_16_ = 1.2 × 10^10^ M^−1^ s^−1^.

While these low O_2_ concentrations hardly affect *g*(H_2_), they do prevent, at long times, the reactions of e_aq_^−^ with itself and with water^62^

17 e_aq_^−^ + H_2_O → H˙ + OH^−^, *k*_17_ = 19 M^−1^ s^−1^

in the bulk of the solutions.

**Fig. 2:**
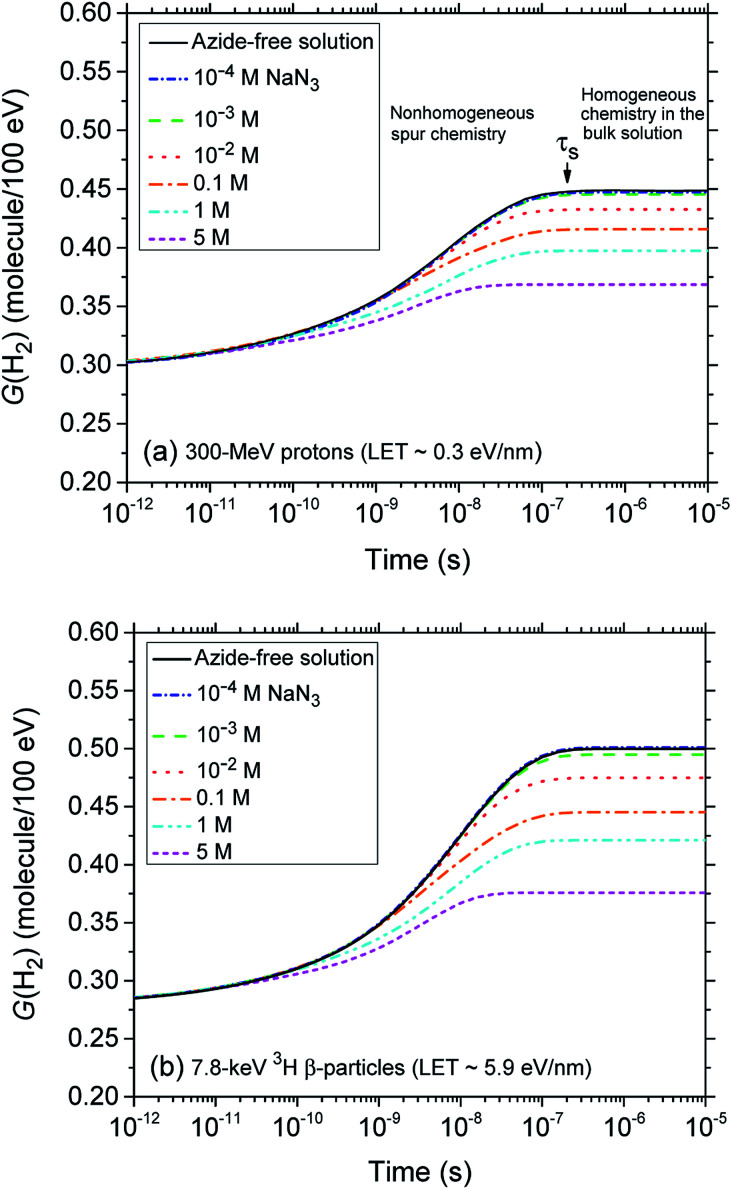
Time evolution of the H_2_ yield (in molecule per 100 eV) for the radiolysis of air-saturated aqueous sodium azide (NaN_3_) solutions by 300 MeV incident protons (which mimic irradiation with ^60^Co γ-rays or fast electrons, LET ∼0.3 eV nm^−1^) (panel a) and by 7.8 keV ^3^H β-particles (LET ∼5.9 eV nm^−1^) (panel b) at neutral pH and 25 °C. Calculations were carried out using our Monte-Carlo track chemistry simulations over the time interval 1 ps to 10 μs. The blue, green, red, orange, cyan, and magenta lines correspond to six different concentrations of N_3_^−^ anions: 10^−4^, 10^−3^, 10^−2^, 0.1, 1, and 5 M, respectively. For both types of radiation, the limiting plateau values of *G*(H_2_) continuously decrease with increasing the concentration of N_3_^−^ ions. For ^60^Co γ/fast electron irradiation, the arrow pointing downwards indicates the time *τ*_s_ ∼0.2 μs required for the changeover from nonhomogeneous spur kinetics to homogeneous kinetics in the bulk solutions, at 25 °C. The black solid line in panels a and b show the kinetics of H_2_ formation in azide-free aerated solutions (shown here for the sake of reference). Finally, the concentration of dissolved oxygen used in the simulations was 2.5 × 10^−4^ M.

Fig. 2 (panels a and b) shows the effect of azide concentration on the kinetics of H_2_ formation over the interval ∼1 ps to 10 μs, as obtained from our Monte Carlo simulations of the radiolysis of aerated neutral pH aqueous solutions of NaN_3_ by ∼300 MeV incident protons and ∼7.8 keV tritium β-electrons at 25 °C. Results are shown for six different concentrations of azide anions, ranging from 10^−4^ to 5 M. As can be seen, for both types of radiation, the time profiles of the H_2_ yields are essentially similar although the magnitude of the *G*(H_2_) values differs. In fact, the simulations show a clear increase in the absolute value of *G*(H_2_) for ^3^H β-electrons compared to ^60^Co γ-rays. As mentioned earlier, this increase in H_2_ yields, when comparing the effects of higher-LET tritium β-radiolysis with the γ/fast electron-radiolysis, is consistent with differences in the initial structure of electron tracks in the two cases. In the short-track geometry of the β-electrons (in contrast with spur geometry), the reactive intermediates are formed in much closer initial proximity, which is favorable to the additional formation of H_2_ through the inter-radical combination reactions (1)–(3).

**Fig. 3:**
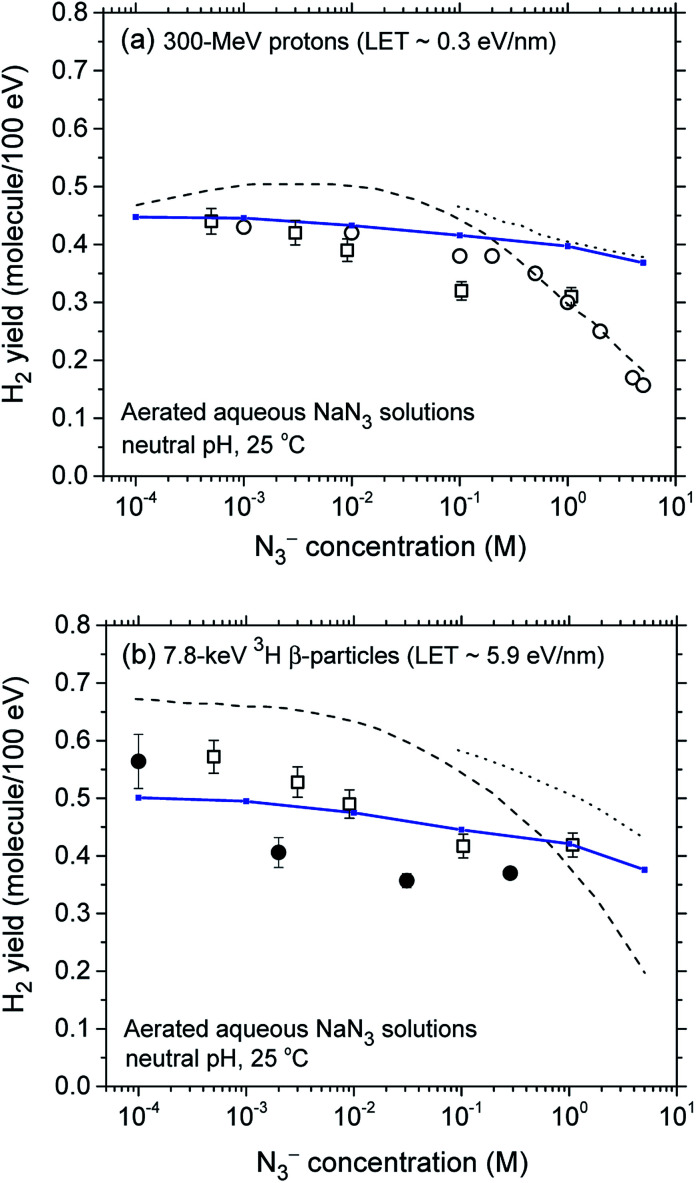
Decrease in the molecular hydrogen yield (in molecule per 100 eV) with concentration of N_3_^−^ ions for 300 MeV incident protons (LET ∼ 0.3 eV nm^−1^) (panel a) and for 7.8 keV ^3^H β-particles (LET ∼ 5.9 eV nm^−1^) (panel b) in the radiolysis of air-saturated aqueous azide (NaN_3_) solutions (neutral pH, 25 °C), calculated from our Monte Carlo simulations over the range of 10^−4^ to 5 M. The blue solid lines show our simulated results (see text). Experimental data for γ and tritium β-particle irradiations: (●), ref. 22; (□), ref. 25; (○), ref. 60. For the sake of comparison, the H_2_ yields calculated from ref. 26 for both types of radiation, assuming that N_3_^−^ scavenges the short-lived precursor to H_2_ with a rate constant of 10^12^ M^−1^ s^−1^ (dashed line) and does not scavenge the short-lived precursor to H_2_ (dotted line), are also shown in the figure.

The decrease in the yield of H_2_ with concentration of N_3_^−^ ions for 300 MeV incident protons and 7.8 keV ^3^H β-electrons in the radiolysis of aerated azide solutions is further illustrated in Fig. 3. The H_2_ yields shown in this figure are the *G*(H_2_) limiting plateau values corresponding to each considered N_3_^−^ concentration, taken from Fig. 2. As can be seen, our simulated yields compare well with the experimental escape yields of Gagnon and Appleby,^22^ Christman,^25^ and Peled *et al.*^60^ obtained for ^60^Co γ and tritium β-particle irradiations. In the case of γ-radiolysis, this agreement is particularly good at low and moderated N_3_^−^ concentrations. However, at concentrations higher than ∼0.5 M, there are significant differences, the experimentally observed H_2_ yields showing a very sharp decrease^73^ compared to the simulation results. This efficiency in reducing the molecular hydrogen produced strongly suggests that the concentration of azide ions is now high enough to allow their reaction with the dry electron (e_dry_^−^) prior to trapping and hydration (*i.e.*, with the precursor to e_aq_^−^), in the subpicosecond physicochemical stage.^33^

Similar findings about the N_3_^−^ scavenging of the short-lived hydrated electron precursor were obtained by Harris and Pimblott^26^ in recent Monte Carlo studies of the ^60^Co γ-radiolysis of azide solutions of concentration greater than 1 M. The present study clearly corroborates their results. Assuming the validity of this hypothesis would imply a (e_dry_^−^ + N_3_^−^) reaction rate constant of ∼10^12^–10^13^ M^−1^ s^−1^ at 25 °C, in agreement with Harris and Pimblott^26,74^ results.

For the case of ^3^H β-particle radiolysis, the effectiveness in lowering *g*(H_2_) at high azide concentration differs considerably from the case of γ-radiolysis. Despite a relatively large dispersion of experimental data,^75^ we do not observe any sharp decrease at concentrations higher than ∼0.1–1 M as we do for γ irradiation. There is only a slight continuous decrease of the yield of H_2_ without any clear supporting evidence that, in this case, N_3_^−^ ions scavenge the short-lived dry electrons. This is consistent with the enhanced contribution of short tracks for the higher LET tritium β-radiolysis as compared to γ radiolysis. Indeed, in this case, the short-track geometry would be competitively more favorable to the subpicosecond recombination reaction of e_dry_^−^ with its nearby parent water cation (H_2_O˙^+^) than to its scavenging by the homogeneously distributed N_3_^−^ ions.

**Fig. 4:**
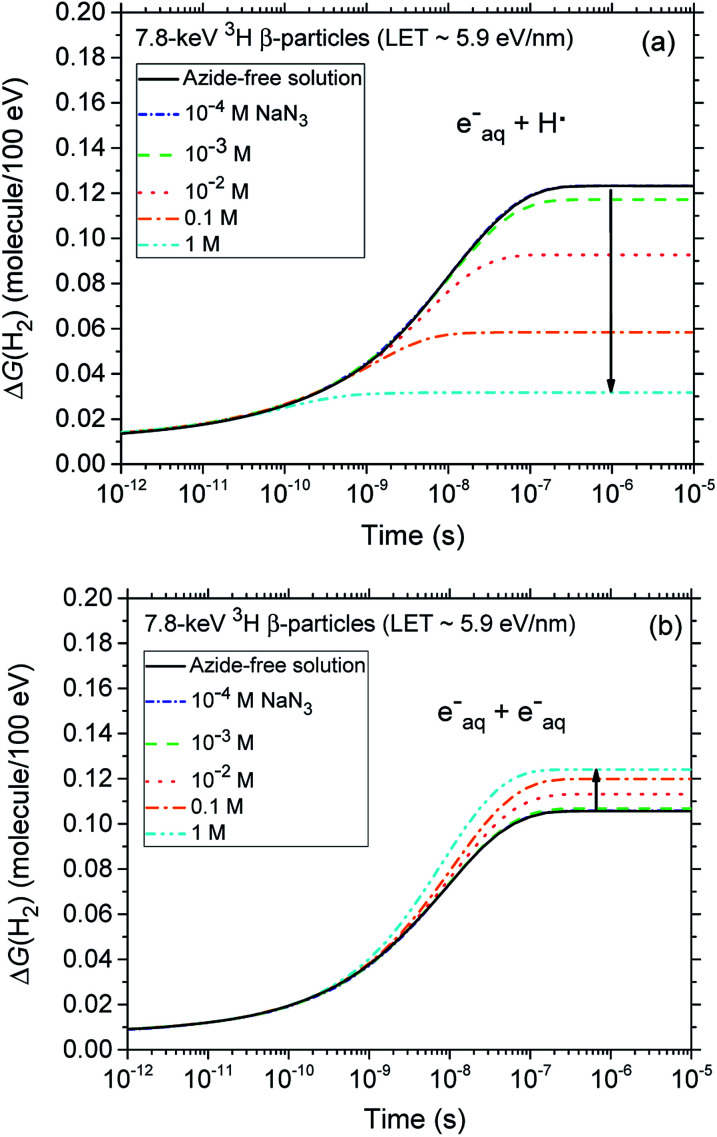
Time dependence of the extents Δ*G*(H_2_) (in molecule/100 eV) of the reactions (e_aq_^−^ + H˙) (panel a) and (e_aq_^−^ + e_aq_^−^) (panel b) that contribute to the formation of molecular hydrogen, calculated from our Monte Carlo simulations of the radiolysis of air-saturated aqueous azide (NaN_3_) solutions (pH neutral, 25 °C) by 7.8 keV ^3^H β-particles (LET ∼ 5.9 eV nm^−1^) in the time interval 1 ps to 10 μs. The blue, green, red, orange, and cyan lines correspond to the five different concentrations of azide anions studied: 10^−4^, 10^−3^, 10^−2^, 0.1, and 1 M, respectively (see text). For the sake of reference, the black lines in panels a and b show the cumulative yield variations Δ*G*(H_2_) of the two reactions (e_aq_^−^ + H˙) and (e_aq_^−^ + e_aq_^−^) that contribute to the formation of H_2_ in azide-free solutions. Finally, the concentration of dissolved oxygen used in the simulations was 2.5 × 10^−4^ M.

A final remark should be made here regarding the origin of the small reduction that is observed, for both types of radiation, in the yields of H_2_ with increasing azide concentration from 10^−4^ up to ∼0.1–1 M. In fact, as shown in Fig. 4, our calculations indicate that the H_2_ production originating from the (H˙ + e_aq_^−^) reaction (1) quickly decreases as the N_3_^−^ concentration increases. This result is of course a clear signature that N_3_^−^ ions readily scavenge H˙ atoms, thus preventing them from contributing to this reaction. By contrast, the formation of H_2_ through the (e_aq_^−^ + e_aq_^−^) reaction (2) should in principle be rather unaffected by the presence of N_3_^−^, N_3_^−^ being highly unreactive towards e_aq_^−^. Actually, it is indirectly because the hydrated electrons that have not reacted with H˙ through reaction (1) become now available to participate to reaction (2). Overall, there is a kind of compensation between the two contributions involved in the H_2_ production, the contribution from reaction (1) dominating slightly.

## 4. Conclusions

Monte Carlo track chemistry simulations have been employed to investigate the scavengeability by azide ions (N_3_^−^) of the molecular hydrogen yield produced in water irradiated with 300 MeV protons (which mimic irradiation with ^60^Co γ rays or fast electrons) and tritium β-electrons at 25 °C. From this study, we clearly show that the formation of H_2_ from ^3^H β-particles is higher than in the case of ^60^Co γ rays, a result that is easily explained by the difference of the structure of radiation tracks. The track structure in the case of ^60^Co γ irradiation is composed of well-separated (spherical) spurs, which contrasts with the short (roughly cylindrical) tracks observed in the case of higher-LET tritium β-electrons. The greater linear energy transfer of ^3^H β-electrons leads to an increased local concentration of reactants. The distance between the primary events is thus much smaller than in the tracks of ^60^Co γ rays. Consequently, we find more molecular products (H_2_ in the case considered in this work) in tritium radiolysis than in γ radiolysis.

Our calculations of the H_2_ yields from γ- and ^3^H β-radiolysis of NaN_3_ solutions show a very good agreement with experiment over a large range of N_3_^−^ concentrations. For ^60^Co γ-radiolysis, however, our H_2_ yields fail to reproduce the sharp decrease that is observed experimentally at high (>1 M) azide concentrations. These results are consistent with previous Monte Carlo simulations that suggested that such a decrease reflected the possibility that low-energy (or “dry”) secondary electrons could be scavenged by N_3_^−^ prior to trapping and hydration in the subpicosecond physicochemical stage. Most interestingly, for ^3^H β-radiolysis, we do not observe any marked decrease in the molecular hydrogen yields at high N_3_^−^ concentrations as we do for γ irradiation. In other words, there is no clear evidence that, in this case, N_3_^−^ ions scavenge the short-lived dry electrons. This is consistent with the enhanced contribution of short tracks for the higher LET ^3^H β-radiolysis as compared to γ radiolysis. Indeed, the short-track geometry is competitively more favorable to the geminate recombination of e_dry_^−^ with their nearby parent water cations than their scavenging by the homogeneously distributed N_3_^−^ ions. In order to further examine these results, we are currently working to introduce this ultra-fast (<1 ps) capture of the dry electron into our simulation models.

In summary, this work, like our previous ones on the subject, provides a strong support for a picture of tritium β-radiolysis in terms of short tracks of high local LET.

## Conflicts of interest

There are no conflicts of interest to declare.

## Supplementary Material
